# The effects of lithium and inflammation on the atherosclerosis of older bipolar patients at high risk for cardiovascular disease

**DOI:** 10.1192/j.eurpsy.2022.1018

**Published:** 2022-09-01

**Authors:** S. Tsai

**Affiliations:** Taipei Medical University, Psychiatry, Taipei, Taiwan

**Keywords:** Lithium, atherosclerosis, older bipolar patients, inflammation

## Abstract

**Introduction:**

Atherosclerosis can result in serious cardiovascular disease (CVD) and is associated with inflammation and psychopharmacological treatment in bipolar disorder.

**Objectives:**

We attempt to investigate the effects of lithium and inflammation on the atherosclerotic development in older bipolar adults at high risk for cardiovascular disease.

**Methods:**

The euthymic out-patients with bipolar I disorder aged over 45 years and concurrent endocrine or cardiovascular disease were recruited to measure their bilateral carotid intima media thickness (CIMT) and circulating levels of lithium, valproate, sTNF-R1, sIL-6R, and lipid profile. All clinical information were obtained by directly interviewing patients and reviewing all medical records.

**Results:**

Forty eight patients with mean 48.3 years old and mean 27.2 years of age at illness onset were recruited. After controlling for the body mass index, multivariate regression analyses showed that older age, lower lithium level, and higher plasma sTNF-R1 level were associated with higher CIMT and collectively accounting for 33.1% of the variance in CIMT. Blood level of low density lipid or valproate has none relationship with CIMT.

**Conclusions:**

Lithium treatment may protect older bipolar patient, even those at high risk for CVD, from atherosclerotic development. Furthermore, persistent inflammatory activation, particularly macrophage activation, may be associated with the accelerating development of atherosclerosis.

**Disclosure:**

No significant relationships.

